# PEGylation of Octreotide Using an α,β-unsaturated-β′-mono-sulfone Functionalized PEG Reagent

**Published:** 2012

**Authors:** Laleh Erfani-Jabarian, Rasoul Dinarvand, Mohammad Reza Rouini, Fatemeh Atyabi, Mohsen Amini, Negar Mohammadhosseini, Abbas Shafiee, Alireza Foroumadi

**Affiliations:** a *Department of Pharmaceutics, Faculty of Pharmacy, Tehran University of Medical Sciences, Tehran, Iran.*; b *Nanotechnology Research Center, Tehran University of Medical Sciences, Tehran, Iran. *; c *Drug Design and Development Research Center, Tehran University of Medical Sciences, Tehran, Iran.*; d *Department of Medicinal Chemistry, Faculty of Pharmacy, Tehran University of Medical Sciences, Tehran, Iran.*; e *Pharmaceutical Sciences Research Center, Tehran University of Medical Sciences, Tehran, Iran.*

**Keywords:** PEGylation, Disulfide bond, Octreotide, Bis-thiol alkylating PEG reagent

## Abstract

PEGylation is a well-established technique utilized to overcome the problems related to the therapeutic applications of peptides and proteins. Reasons for the PEGylation of these biological macromolecules include reducing immunogenicity, proteolytic degradation and rapid clearance from blood circulation. Octreotide is an octapeptide analogue of naturally-occurred somatostatin. This peptide has elimination half-life of less than 2 h that requires frequent daily subcutaneous or intravenous administration. To address this issue, octreotide modification was investigated using bis-thiol alkylating PEG reagent. The required bis-thiol alkylating reagent (V) was prepared from commercially available 4-acetyl benzoic acid in five steps. Octreotide disulfide bond was mildly reduced to liberate the two cysteine sulfur atoms followed by bis-alkylation to form PEGylated peptide. The PEG modification process was monitored through the reverse phase HPLC and ^1^H-NMR analysis. According to the HPLC chromatograms of PEGylation reaction, the peak with 30 min retention time was identified to be PEG-octreotide. In addition, ^1^H-NMR analysis showed a 7.44% degree of PEG substitution.

## Introduction

Octreotide, an octapeptide analogue of naturally occurring somatostatin, is clinically used for the treatment of acromegaly and endocrine tumors (1, 2). Recently, therapeutic potential of this peptide has been extended to non-endocrine tumors, particularly primary hepatocellular carcinoma (3). Octreotide-therapies suffer from a relatively short half-life of the products in circulation that requires frequent daily subcutaneous or intravenous administration. To address this issue, polyethylene glycol (PEG) modification (PEGylation) was considered for the possible half-life and bio-distribution optimization using polyethylene glycol covalent attachment. 

PEGylation is one of the several techniques which have been successfully applied to proteins and peptides to improve their pharmacokinetic and pharmacodynamic properties. The beneficial effects of PEGylation can be achieved through increasing stability and solubility of the PEGylated product, reducing renal clearance and clearance via antibodies as well as reducing proteolytic degradation (4). 

*N*-terminal PEGylation of octreotide has been disclosed in some literatures to prevent acylation through polyester polymers such as polylactic acid and polylactic-co-glycolic acid which are used in octreotide sustained release formulations. As octreotide has two PEGylation sites of *N*-terminus (Phe^1^ and Lys^5^), following *N*-terminal PEGylation, heterogeneous mixture of PEGylated species with different binding sites and PEG numbers (mono-PEG-Phe^1^-, mono-PEG-Lys^5^- and di-PEG-octreotide) were produced (5-8). In addition, amine- specific PEG reagents such as active esters, carbonates and isocyanates have a short half-life in aqueous solution, and aldehydes are inefficient for conjugating proteins; therefore, a large stoichiometric excess of these reagents is required. These characteristics often cause difficult purification procedures to remove the excess PEG reagent and the unwanted by-products (9). 

To prepare a homogeneous PEGylated peptide, the site-specific conjugation of PEG using both the sulfur atoms in the disulfide bond has been reported in a number of papers (9-12). An accessible disulfide bond was reduced to liberate the two cysteine sulfur atoms without disturbing the protein’s tertiary structure. PEGylation was achieved with a bis-thiol alkylating PEG reagent that undergoes the conjugation to form a three-carbon bridge. The two sulfur atoms were re-linked with PEG selectively conjugated to the bridge (10-12). 

**Figure 1:**
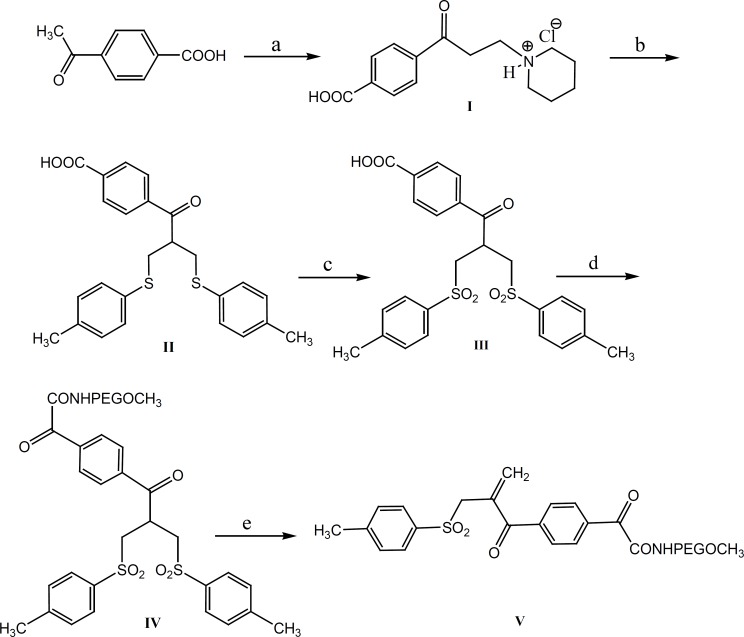
Reaction conditions: a: para formaldehyde, piperidine hydrochloride; b: formalin, piperidine, 4-methylbenzenethiol; c: Oxone; d: DCC, 4-(dimethylamino) pyridine, *O*-(2-aminoethyl)-*O*´-methyl poly(ethylene glycol); e) sodium phosphate buffer, pH 7.8

**Figure 2:**
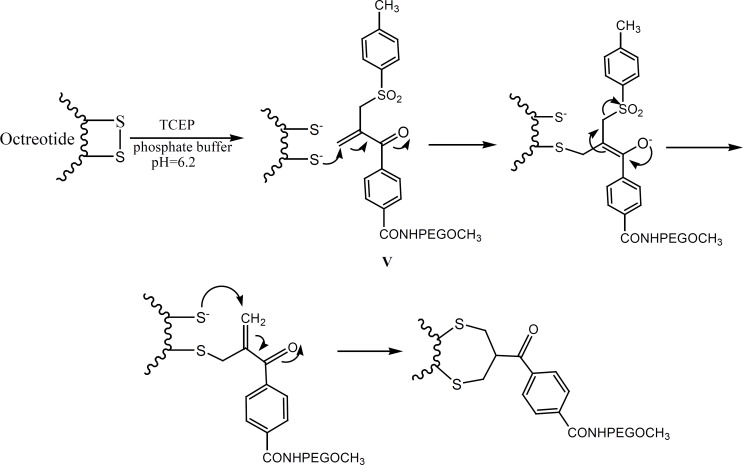
Plausible mechanism for the PEGylation of octreotide by compound V (adopted from reference 10).

The aim of the present study has been to investigate the PEGylation of octreotide using *α,β*-unsaturated-*β*′-mono-sulfone (V**, **Figure 1). As a matter of fact, compound V is a bis-thiol alkylating PEG reagent which reacts with both thiol groups resulted from the reduction of octreotide disulfide bond (Figure 2). The conjugation reaction has been monitored through the reversed-phase HPLC method. 

## Experimental

*Chemicals*

All chemicals were obtained from Sigma- Aldrich and Merck chemical companies. Octreotide acetate (H2N-DPhe-Cys-Phe- DTrp-Lys-Thr-Cys-Throl, Mw = 1019.26) was obtained from Hybio Pharmaceutical company. TLC analyses were performed on a 3-10 cm aluminum sheet pre-coated with silica gel 60-254 (Merck). Melting points were taken on Kofler hot stage apparatus. The pH measurements were performed using Metrohm 827 instrument and RP HPLC set up was Agilent 1200 series with Waters C18 column (Dimension: 250 × 460 mm, Particle Diameter: 5 microns). Amicon Ultra-4 Centrifugal Filters were used for final purification. 

*Experimental protocols*

1-[3-(4-Carboxy-phenyl)-3-oxo-propyl]- piperidinium hydrochloride (I) 

A mixture of absolute ethanol (30 mL), 4-acetylbenzoic acid (5.0 g), piperidine hydrochloride (3.7 g), concentrated HCl (0.3 mL) and paraformaldehyde (2.74 g) was refluxed at 105°C for 10 h. After 4 h of reflux, additional paraformaldehyde (2.74 g) was added to the mixture and the reaction was continued for 6 h. Fifteen mL of acetone was added and the precipitates were collected. The obtained white solid was kept in a vacuum oven at room temperature overnight (11). These procedure afforded compound I, yield 42%; mp 217-218ºC; IR: υmax (KBr) 3459 (NH+), 2552-3200 (OH), 1702 cm-1 (C=O); 1H-NMR (DMSO-d6): 2.4-2.8 (m, 6H) 3.2 (s, 4H), 3.6 (t, 2H), 4.0 (t, 2H), 8.8 (bs, 4H, aromatic).

*4-(3-(p-Tolylthio)-2-(p-tolylthiomethyl) propanoyl) benzoic acid (II)*

A mixture of absolute ethanol (12 mL), methanol (8 mL), compound I (3.5 g), 4-methyl benzenethiol (2.91 g), piperidine (0.5 mL) and 37% aqueous formaldehyde (3.5 mL) was stirred at gentle reflux at 105°C for 1 h. Then, while the reaction solution was still being stirred, 37% aqueous formaldehyde (3.5 mL) was added and the mixture was refluxed for an additional 3 h. The mixture was cooled to room temperature and concentrated in-vacuo to obtain a creamy white residue. Methanol (10 mL) was added to the residue and heated to be dissolved. The solution was kept at refrigerator (4°C) for 12 h; the crystallized compound was filtered (11). Yield 70%; mp 130-131ºC; IR: υmax(KBr) 2524-3100 (OH), 1770 cm-1 (C=O); 1H-NMR (CDCl3): 2.36 (s, 6H), 3.16-3.31 (m, 4H), 3.85 (q, 1H), 7.05 (d, 4H, J = 8.4 Hz), 7.13 (d, 4H, J = 8.4 Hz), 7.64 (d, 2H, J = 8.5 Hz), 8.07 (d, 2H, J = 8.5 Hz).

*4-(3-Tosyl-2-(tosylmethyl) propanoyl) *benzoic acid (III)

To a mixture of compound II (3.0 g) in methanol-deionized water solution (1:1v/v, 100 mL) 25.34 g of Oxone® was added and the resulting mixture was stirred at room temperature for 24 h. The mixture was extracted through chloroform (2 × 50 mL). The organic phase was washed with 100 mL of brine solution followed by drying on sodium sulphate. The solution was concentrated in-vacuo and the obtained solid was kept in a vacuum oven at room temperature overnight (11).

Yield 86%; mp 140-141°C; IR: υmax(KBr) 2533-3150 (OH), 1682 (C=O), 1277 cm-1 (SO2); 1H-NMR (CDCl3): 2.49 (s, 6H), 3.48-3.66 (m, 4H), 4.40 (q, 1H), 7.37 (d, 4H, J = 8.0 Hz), 7.70-7.73 (m, 6H), 8.10 (d, 2H, J = 8.4 Hz).

*N-(MethoxyPEG)-4-(3-tosyl-2-(tosylmethyl) propanoyl) benzamide (IV)*

A solution of O-(2-Aminoethyl)-O´-methyl poly (ethylene glycol) (5000 g/mol, 0.5 g), compound III (0.055 g), N, N-dicyclohexylcarbodiimide (DCC) (0.021 g) and 4-dimethyl-aminopyridine (DMAP) (0.0024 g) in dichloromethane/tetrahydrofuran (1:1 v/v) was allowed to be stirred at room temperature for 72 h under dry condition. Dichloromethane /tetrahydrofuran mixture was removed under the reduced pressure; the viscous crude product residue was dissolved in a mixture of ether/hexane (1:1 v/v). The flask was then placed in a dry-ice bath for 5 min to precipitate the product. After filtering and drying in vacuum, compound IV was obtained in high yield (11).

Yield 90%; mp 227-228ºC; IR: υmax(KBr) 3326(NH), 1626(CO), 1243cm-1 (SO2); 1HNMR (CDCl3): 2.49 (s, 6H), 3.38 (s, 3H, CH3OPEG), 3.44-3.84 (m, OCH2CH2O and 4H, CH2SO2), 4.3 (m, 1H, CHCO), 7.35(d, J = 8.2Hz), 7.68(d, J = 8.2Hz), 7.64(d, J = 8.4 Hz), 7.81(d, J = 8.3Hz).

*PEGylation of octreotide*

Compound IV (0.7 mg, 5500 g/mol) was incubated in 50 mM sodium phosphate buffer (1 mL) with pH of 7.8, for 48 h to obtain the N-(methoxyPEG)-4-(2-(tosylmethyl) acryloyl) benzamide (V) (10). Octreotide (0.156 mg/mL, 1 mL) in 50 mM sodium phosphate buffer with pH of 6.2, was disulfide-reduced using 0.043 mg of tris-(2-carboxyethyl) phosphine hydrochloride for 1 h at ambient temperature. The pH of the solution containing compound V was adjusted to 6.2 using 1N HCl and added to the disulfide-reduced octreotide reaction mixture. The reaction mixture was gently shaken and left overnight at 4°C for 24 h. Unreacted thiols of disulfide-reduced octreotide backed to their disulfide bonds using 50 μL of 50 mM glutathione reoxidizing solution after 10 h at 4 °C. The purification of PEG-octreotide conjugate was performed using centrifugal ultra-filter at 4000 × g centrifugal force for 10 minutes. The concentrated solution was then subjected to freeze-drying. The compound V formation monitoring and the purified PEG-octreotide conjugate analysis was performed through the reverse phase HPLC setup using a C18 column (5 μm, 250 × 4.6 mm), (acetonitrile:water (25:75) + 0.1% v/v trifluoroacetic acid) solvent system with a flow rate of 1 mL/min at room temperature and UV detection at 215 nm. 

## Results and Discussion

Our approach to octreotide PEGylation involves the mild reduction of disulfide bond, followed by the reaction with the bis-thiol-specific PEG reagent. The required bis-thiol alkylating reagent (V) was prepared from commercially available 4-acetylbenzoic acid in five steps (10) (Figure 1). 

**Figure 3:**
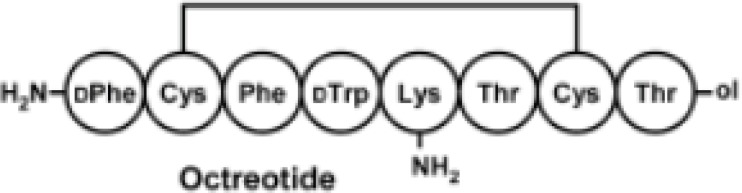
Structure of octreotide

Among eight amino acids of octreotide, six of them are connected through a disulfide bond to form a ring (Figure 3). The disulfide bond reduction of octreotide was accomplished by means of equimolar TCEP-HCl, with pH of 6.2, at ambient temperature. The reduction at higher pH values caused the peptide denaturation. The pure compound IV undergoes the elimination of toluene sulfinic acid to give the compound V at pH values of 7.8 (9-10). Compound V is a bis-thiol alkylating PEG reagent which reacts with both thiol groups resulted from the reduction of octreotide disulfide bond (Figure 2).

**Figure 4:**
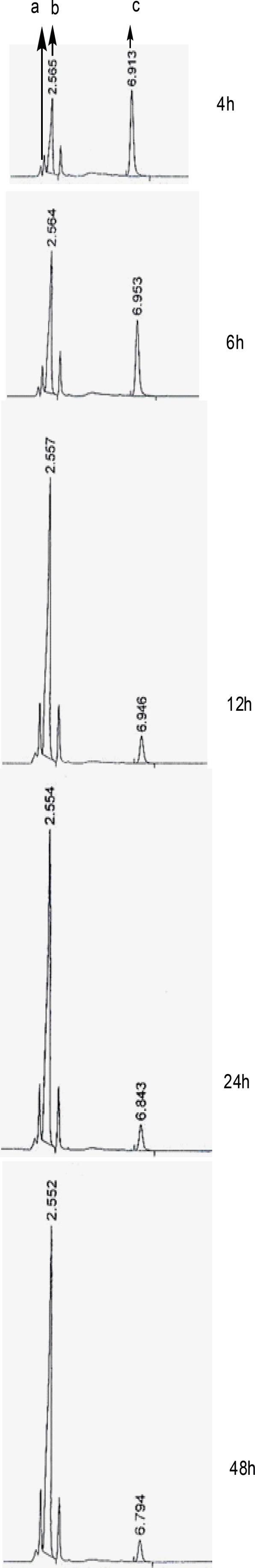
RP-HPLC chromatograms for the elimination of toluenesulfinic acid from compound IV to give V (sampling was performed at 4, 6, 12, 24 and 48 h after starting the reaction).

**Figure 5:**
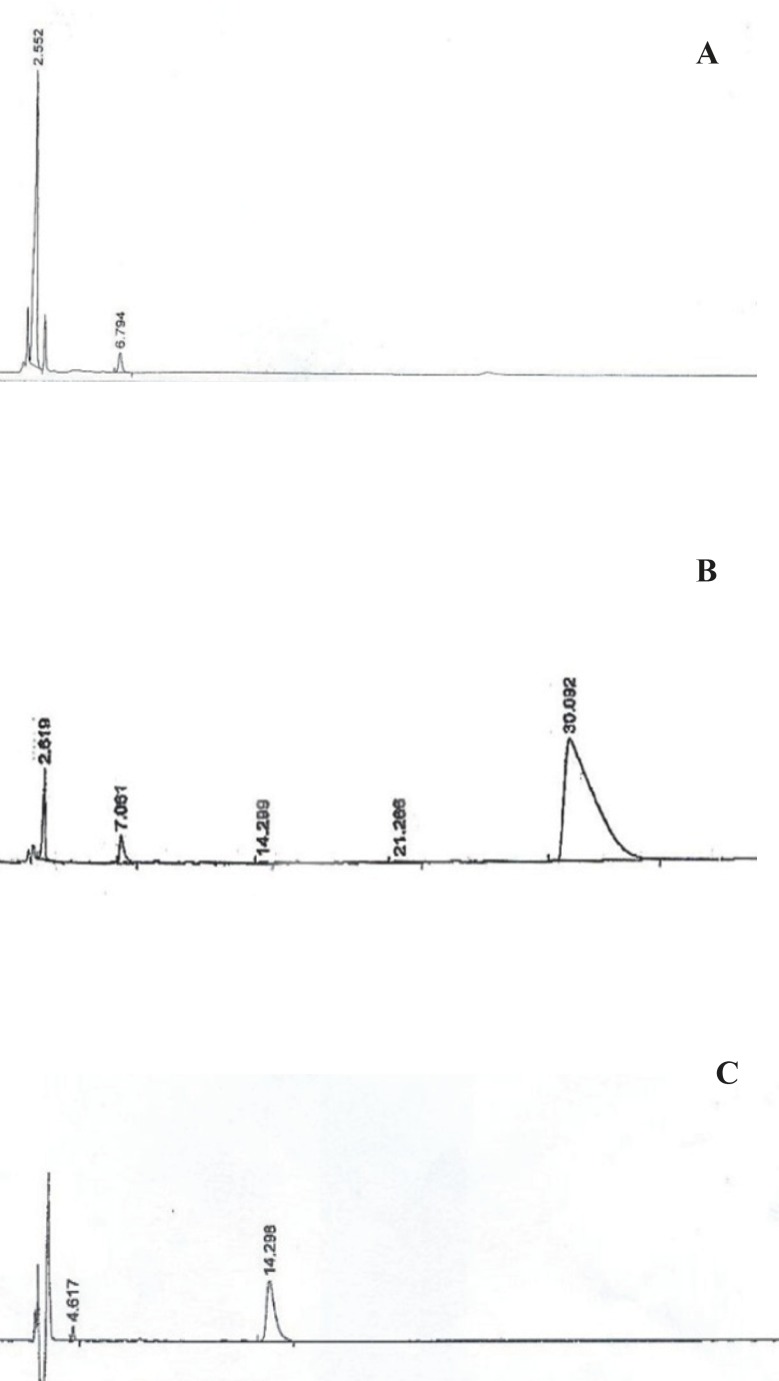
RP-HPLC chromatograms of PEG-octreotide-coupling reaction; (A) Compound formation reaction after 48 h, (B) Coupling reaction: after recycling unreacted octreotide disulfide bond (C) Octreotide standard

The elimination reaction was monitored by reverse phase HPLC method. Figure 4 shows the chromatograms of the reaction mixture, exhibiting 3 peaks: retention times of approx. 2.1 (a) 2.5 (b) and 6.8 (c) in that order. The first peak (a) was identified to be toluene sulfinic acid, the peaks (b) and (c) are related to compounds V and IV, respectively. Due to the instability of disulfide bond of reduced octreotide at pH = 7.8, the pH of solution containing compound V was adjusted to 6.2 before coupling the reaction. The coupling process was monitored through the reversed phase HPLC method (Figure 5).

**Figure 6:**
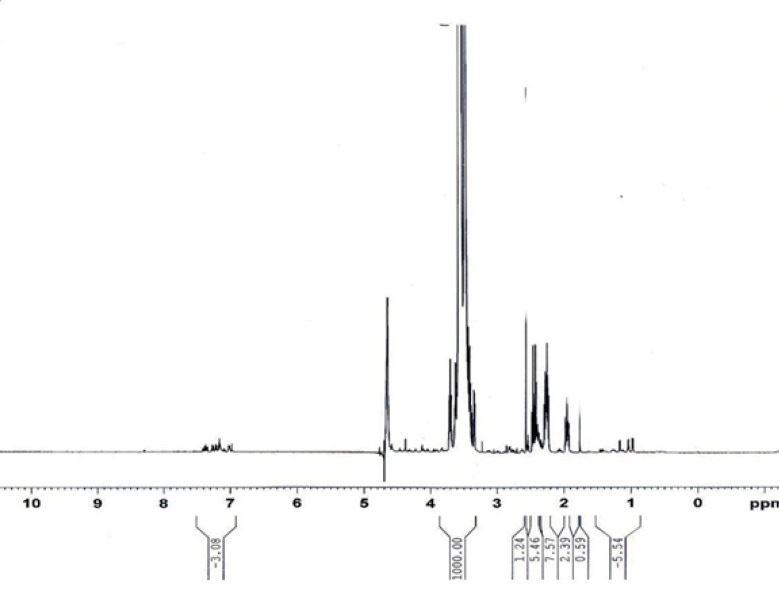
1H-NMR spectrum of PEG-octreotide

 Chromatogram A represents the elimination reaction of compound IV after 48 h*. *The additional peak appeared at 30 min of retention time in chromatogram B was identified to be PEG-octreotide and the peaks at the retention times of 14.289 and 2.619 are related to the unreacted octreotide (according to the standard peak in chromatogram C) and compound V, respectively. ^1^H-NMR analysis of PEG-octreotide is presented in Figure 6. 

The peaks at 3.5-3.7 were attributed to the methylene groups of PEG. The aromatic peaks at 7.00-7.50 ppm were used for the percentage of PEGylation. The comparing area under the curve of peaks at aromatic region and area under the curve of PEG’s protons showed 7.44% degree of substitution.

The coupling reaction of PEG reagent and octreotide was completed in 72 h and simply monitored through the reversed phase HPLC method. The present PEG-octreotide hybrid is promised to show greater circulation half-life and stability toward octreotide; however, further investigations are needed to prove this expectation.
